# Supplementary material for: Discovery of 7-Azanorbornane-Based Dual Agonists for the Delta and Kappa Opioid Receptors through an In Situ Screening Protocol
Source: Molecules. 2023 Oct 3;28(19):6925. doi: 10.3390/molecules28196925 (PMC10574725; doi:10.3390/molecules28196925)

Supporting Information

Discovery of 7-azanorbornane-based dual agonists for the delta and kappa opioid receptors through an *in situ* screening protocol

Fumika Karaki, Taro Takamori, Koumei Kawakami, Sae Sakurai, Kyoko Hidaka, Kei Ishii, Tomoya Oki, Noriko Sato, Nao Atsumi, Karin Ashizawa, Ai Taguchi, Asuka Ura, Toko Naruse, Shigeto Hirayama, Miki Nonaka, Kanako Miyano, Yasuhito Uezono and Hideaki Fujii

Contents

Supplemental figures and tables··························1

^{1}H and ^{13}C NMR spectra of the triazoles··············4

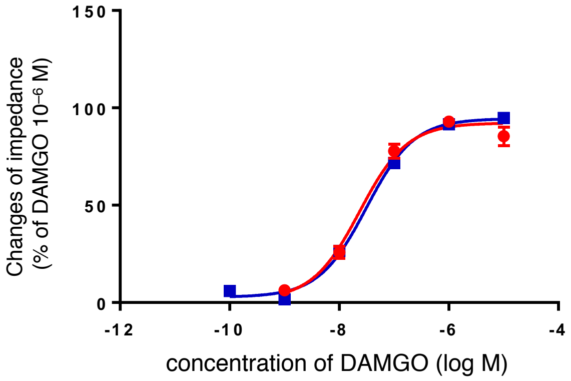

Figure S1. Concentration-dependent effect of DAMGO on the MOR-expressing cells in the presence (red line) and absence (blue line) of the click catalysts (copper sulfate: 1 mol%, sodium ascorbate: 5 mol%).

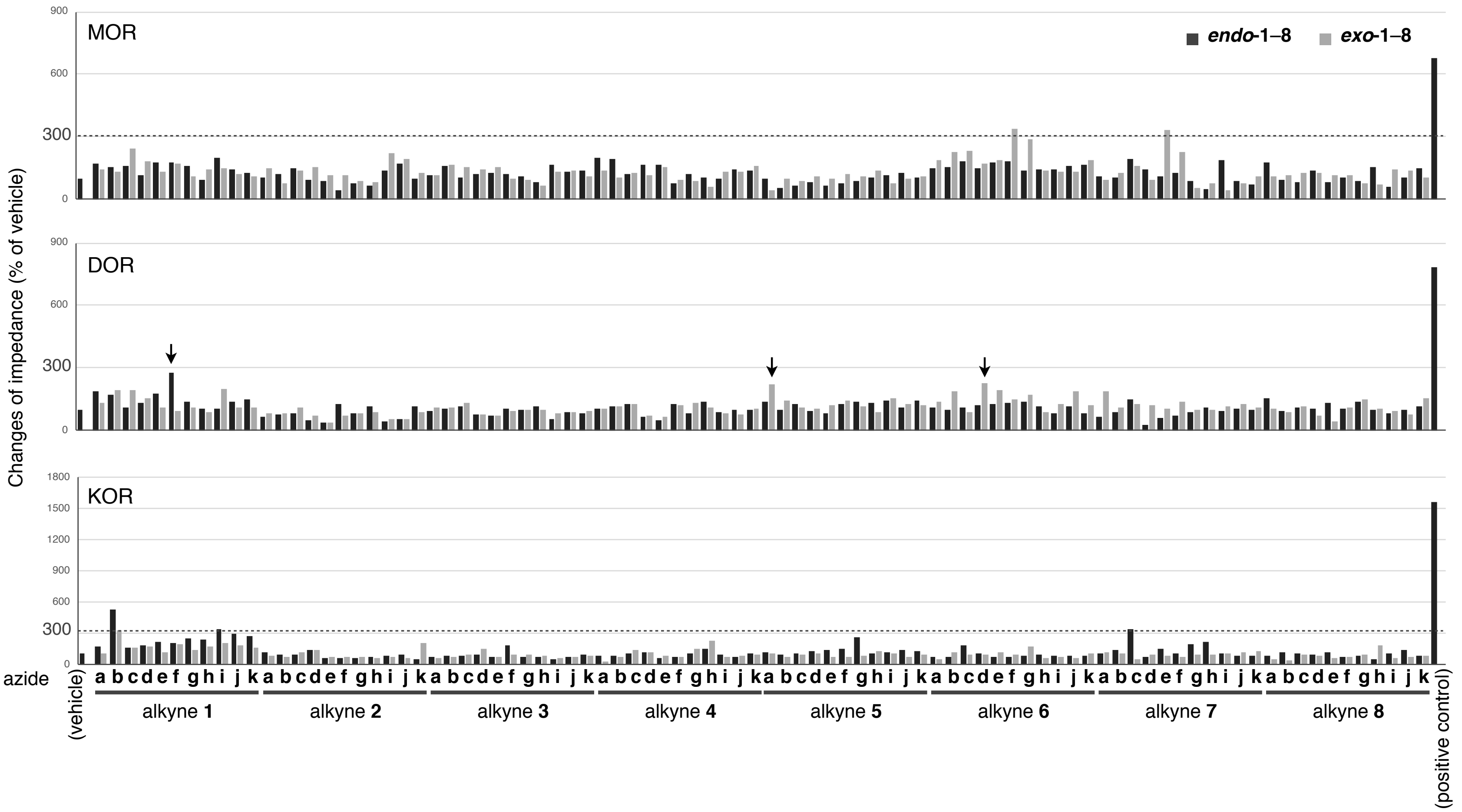

Figure S2. The results of the first screening assays (n = 2 × 1). The concentrations of the triazoles should be 10 μM, assuming that the reaction yield was 100%. The positive controls are DAMGO (for MOR), SNC80 (for DOR), and (–)-U-50,488H (for KOR), and they were treated at a concentration of 10 μM.

Table S1. The E_{max} and $logEC_{50}$ values of the agonistic activities. N.D. = not determined

	DOR		KOR	
	E_{max} (%)	$logEC_{50}$	E_{max} (%)	$logEC_{50}$
positive control	100	–8.46	100	–8.45
***exo*-7e**	90.9	–5.96	84.7	–5.75
***endo*-1b**	60.1	–5.42	82.9	–5.83
***endo*-1i**	N.D.	N.D.	N.D.	N.D.
***endo*-7c**	71.0	–6.36	79.2	–5.40
***endo*-1**	N.D.	N.D.	72.4	–6.75
***exo*-1**	N.D.	N.D.	N.D.	N.D.
***exo*-5**	N.D.	N.D.	N.D.	N.D.
***endo*-7**	N.D.	N.D.	N.D.	N.D.

^{1}H and ^{13}C NMR spectra of the triazoles

^{1}H (400 MHz, DMSO-d_6) and ^{13}C (100 MHz, CD_3OD) NMR spectra of ***endo*-1b**

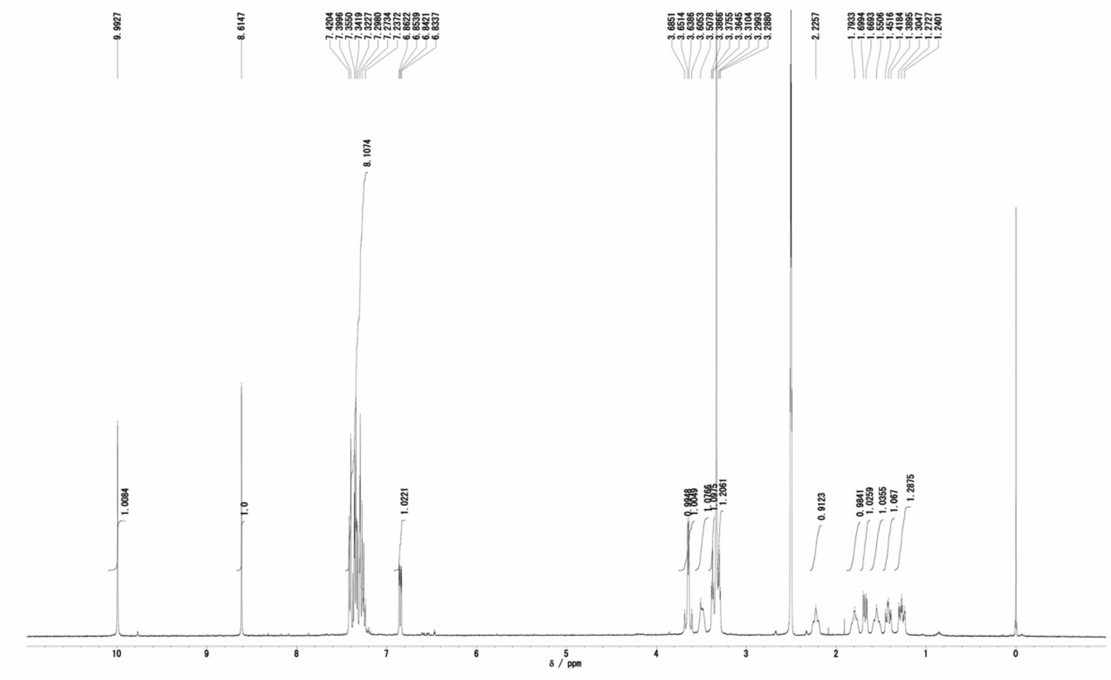

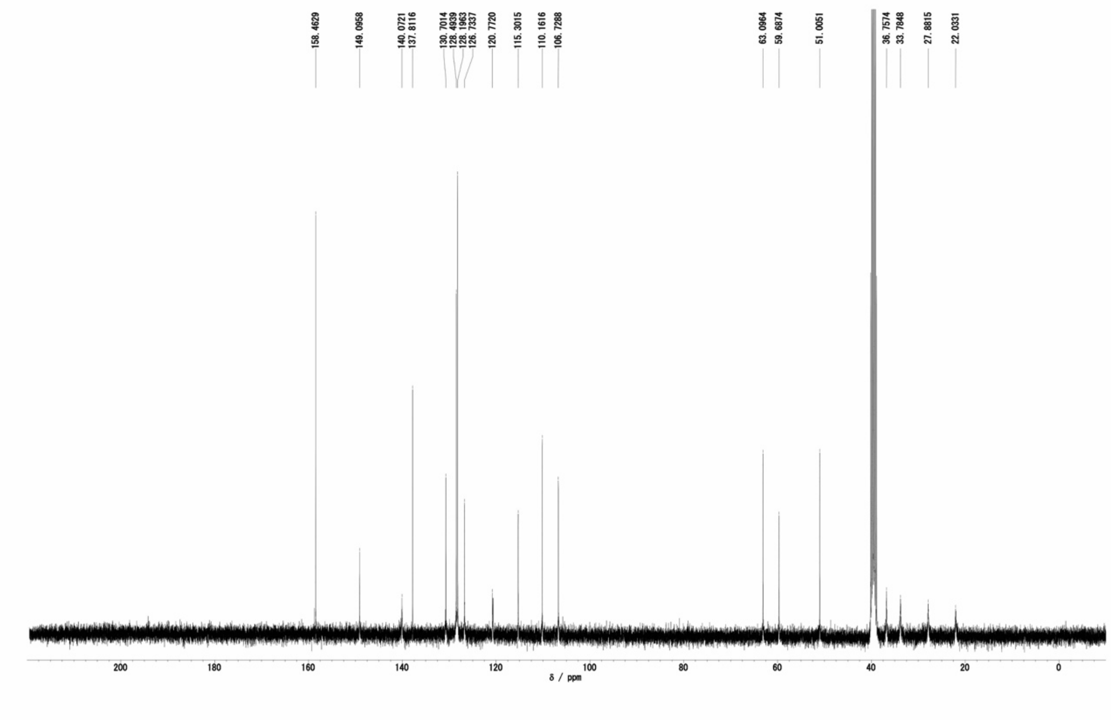

^{1}H (400 MHz, $CDCl_3$) and ^{13}C (100 MHz, DMSO-d_6) NMR spectra of ***exo*-1b**

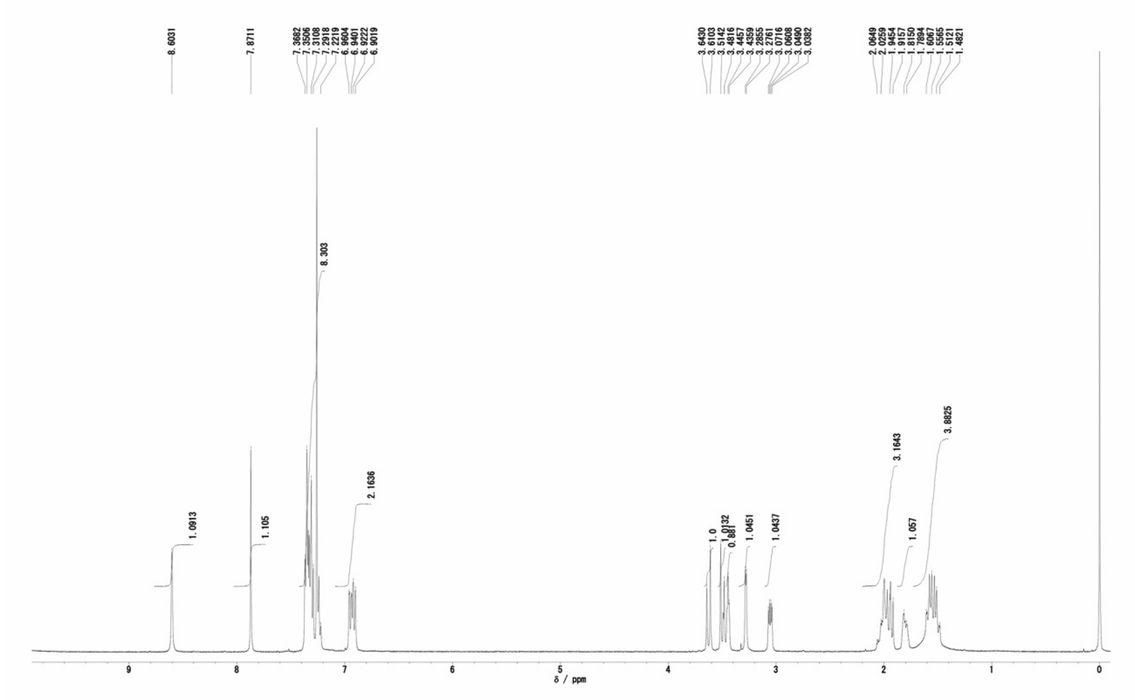

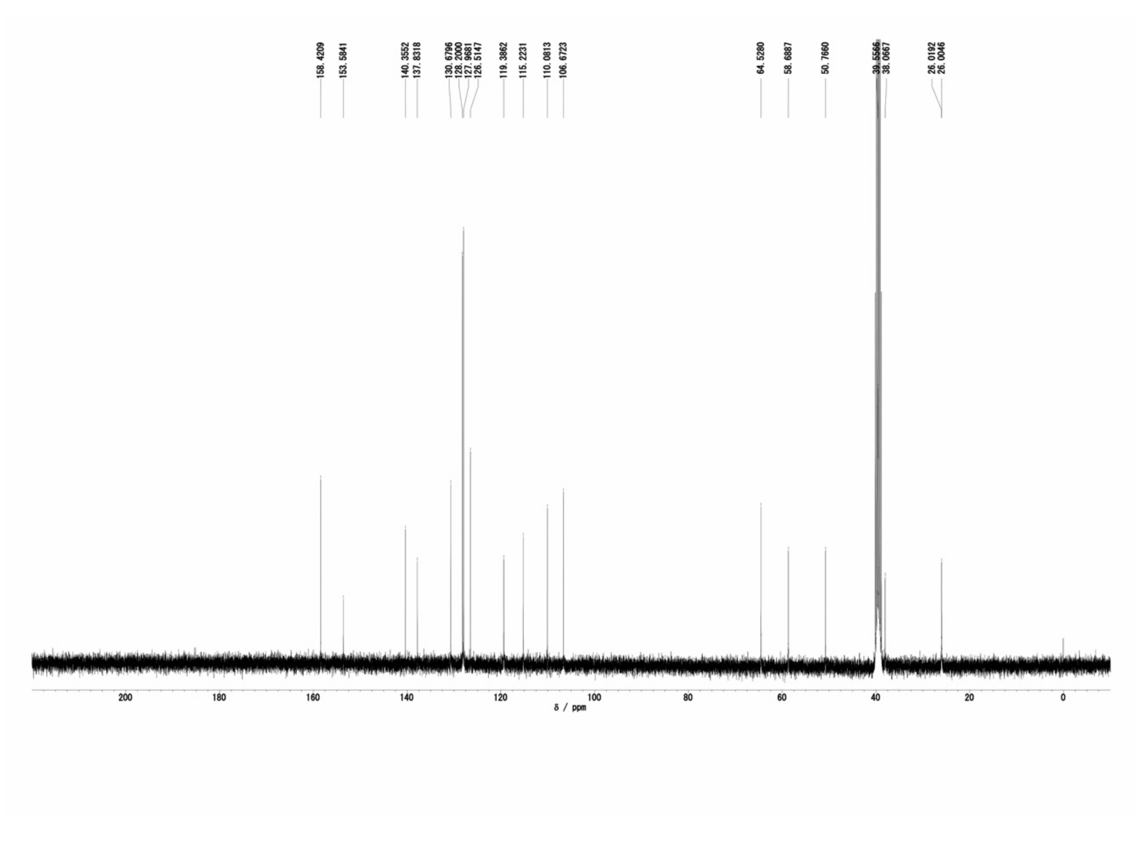

^{1}H (400 MHz) and ^{13}C (100 MHz) NMR spectra of ***endo*-1f** ($CDCl_3$)

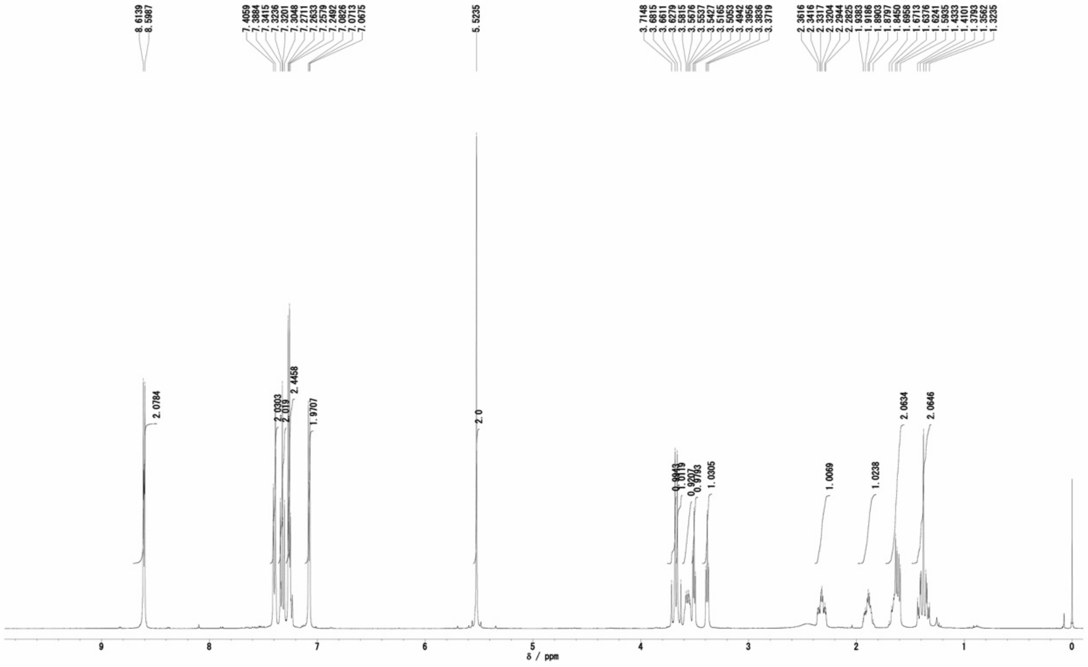

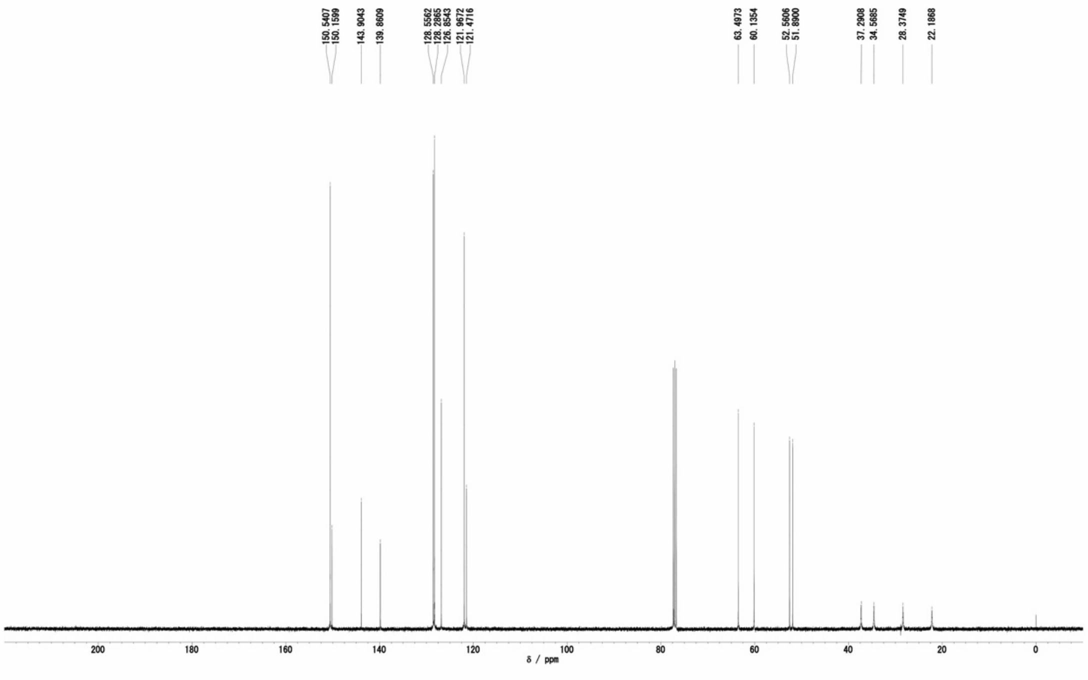

^{1}H (400 MHz) and ^{13}C (100 MHz) NMR spectra of ***endo*-1i** ($CDCl_3$)

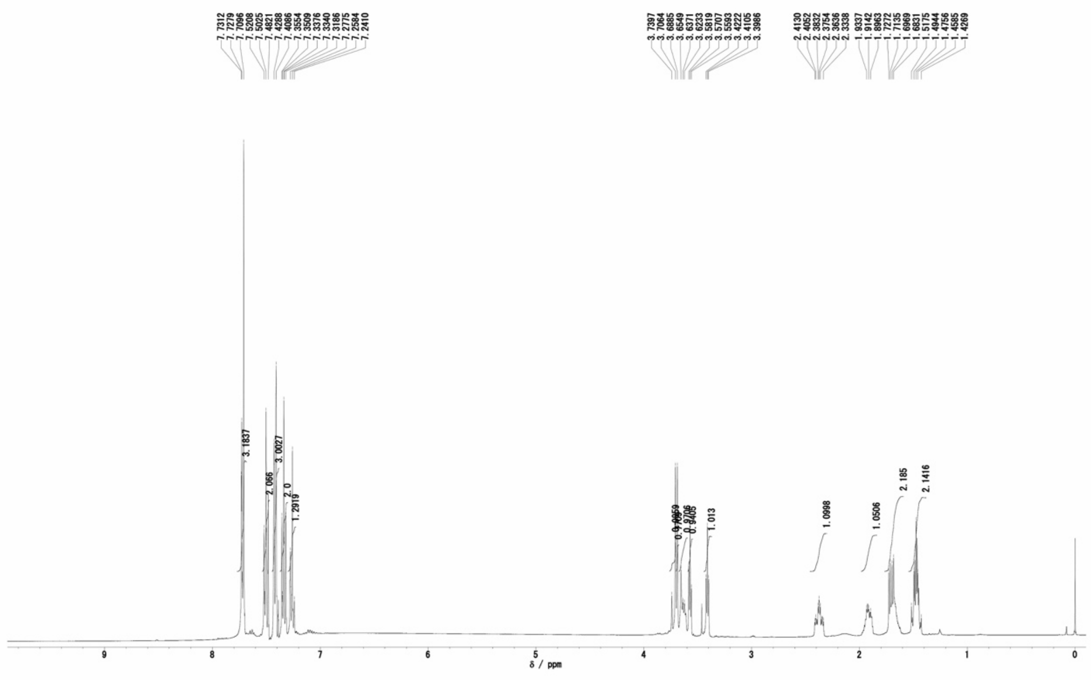

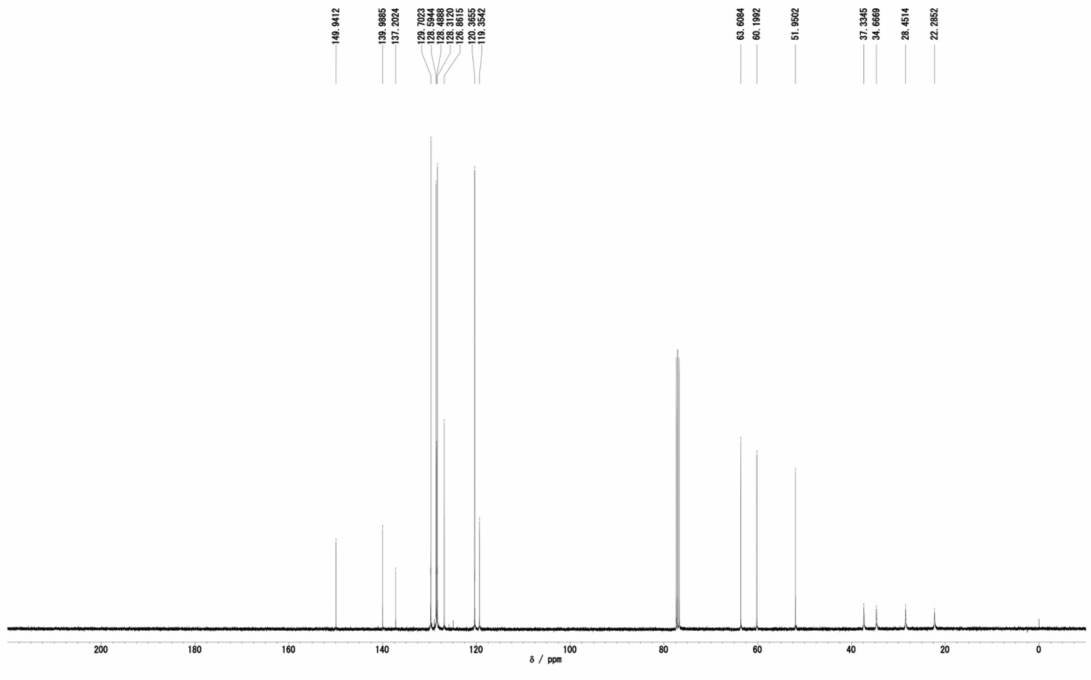

^{1}H (400 MHz) and ^{13}C (100 MHz) NMR spectra of ***exo*-5a** ($CDCl_3$)

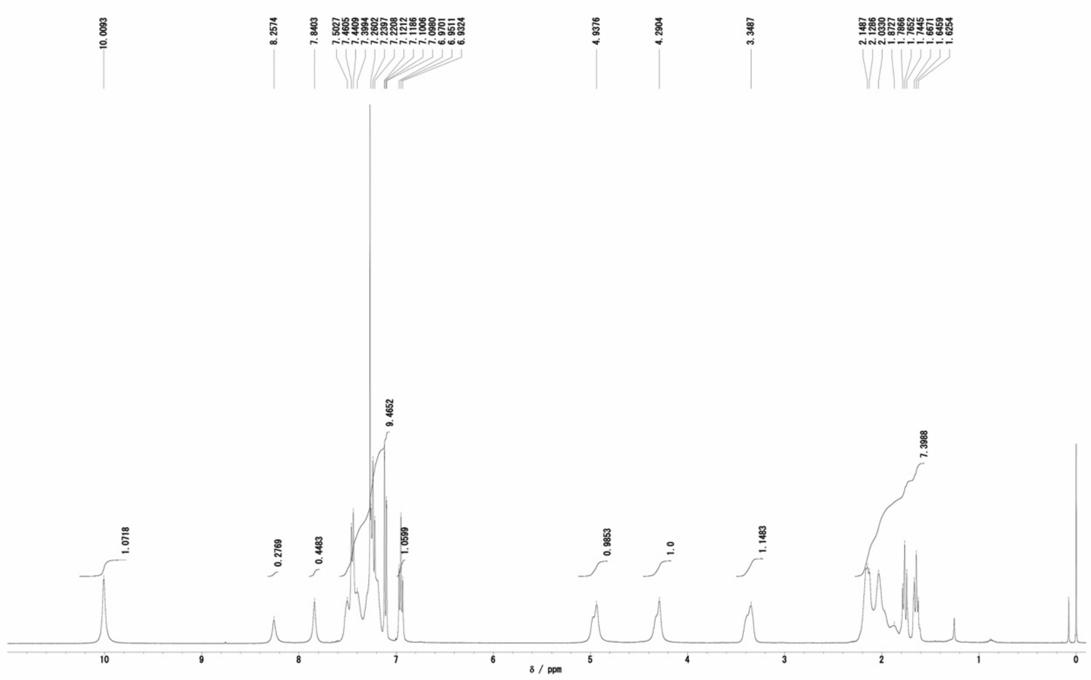

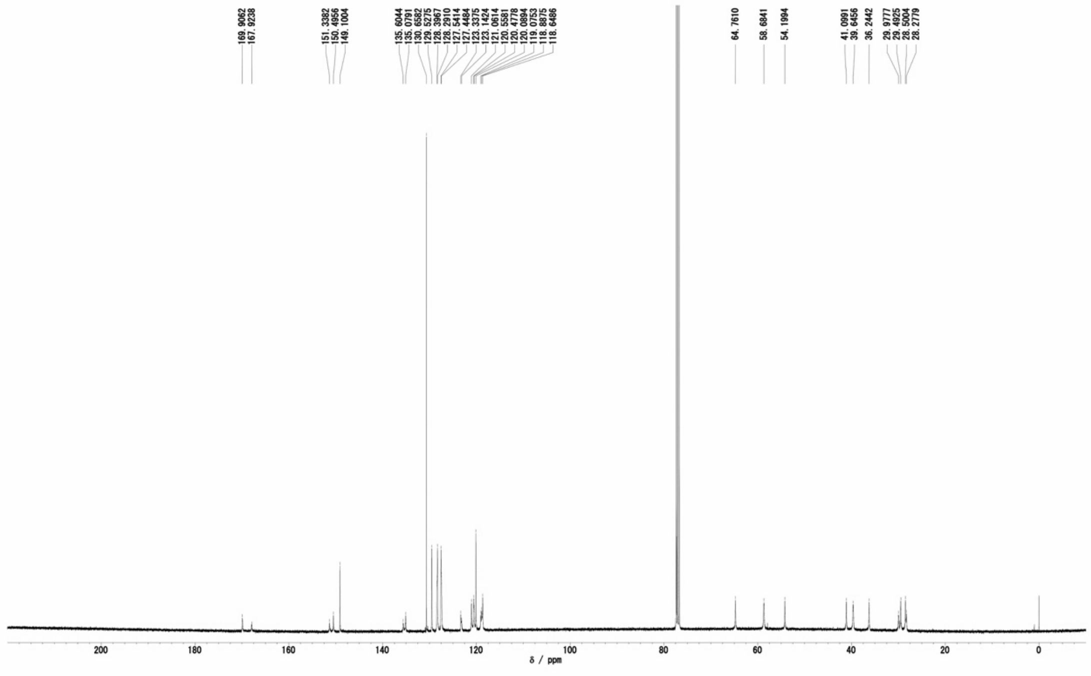

^{1}H (400 MHz) and ^{13}C (100 MHz) NMR spectra of ***exo*-6d** ($CDCl_3$)

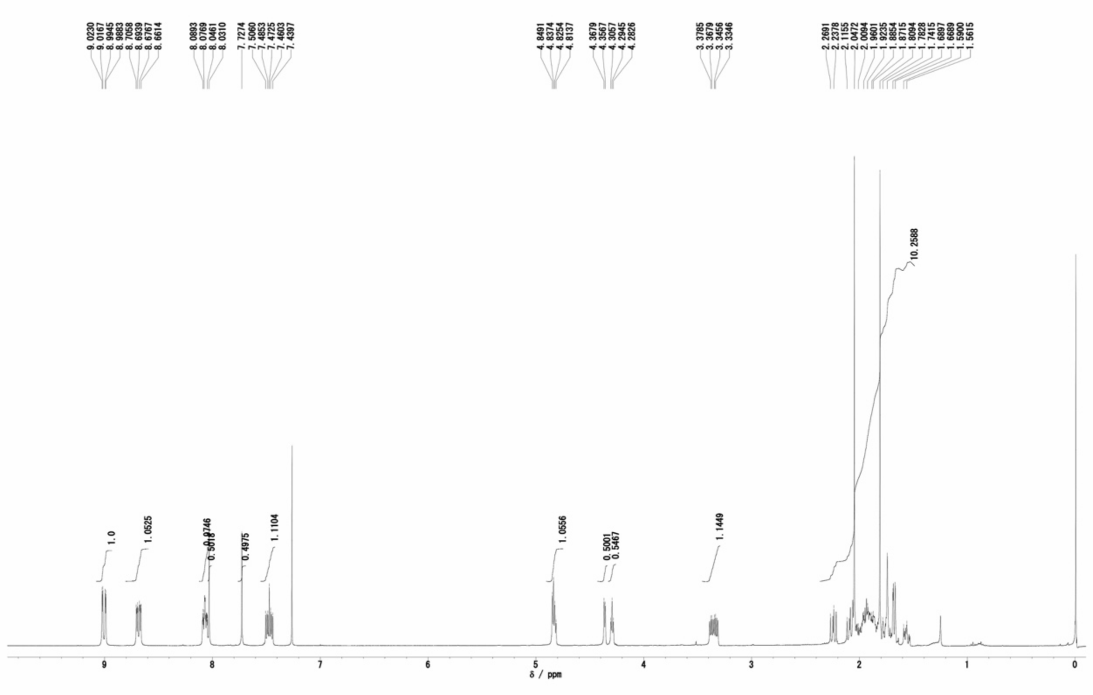

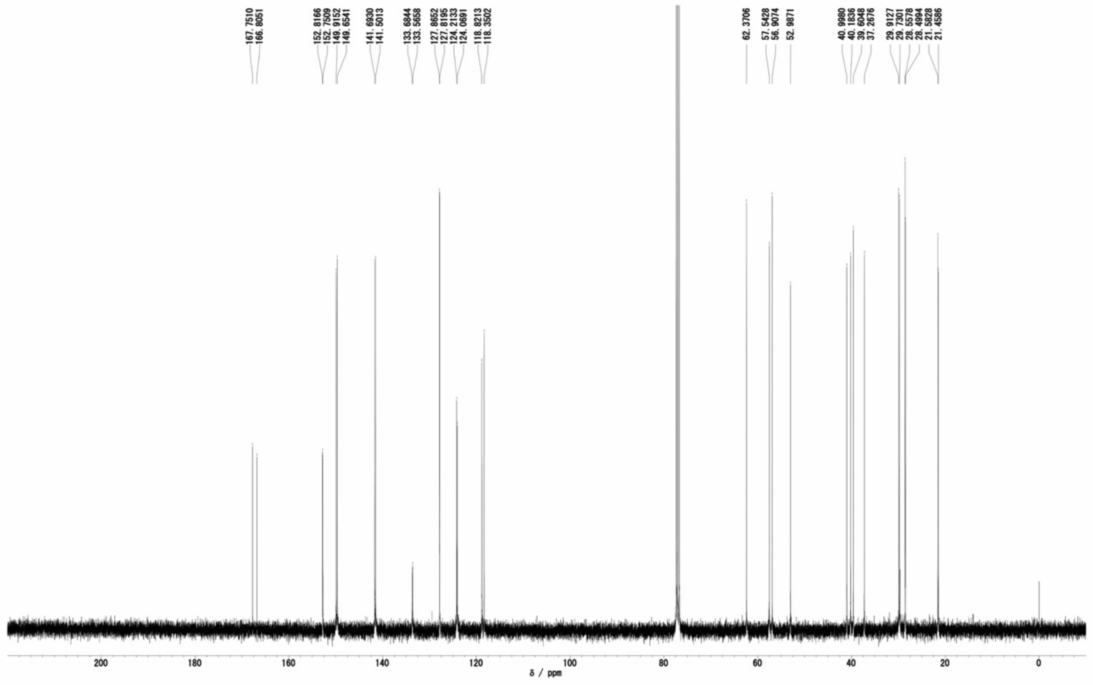

^{1}H (400 MHz) and ^{13}C (100 MHz) NMR spectra of ***exo*-6f** ($CDCl_3$)

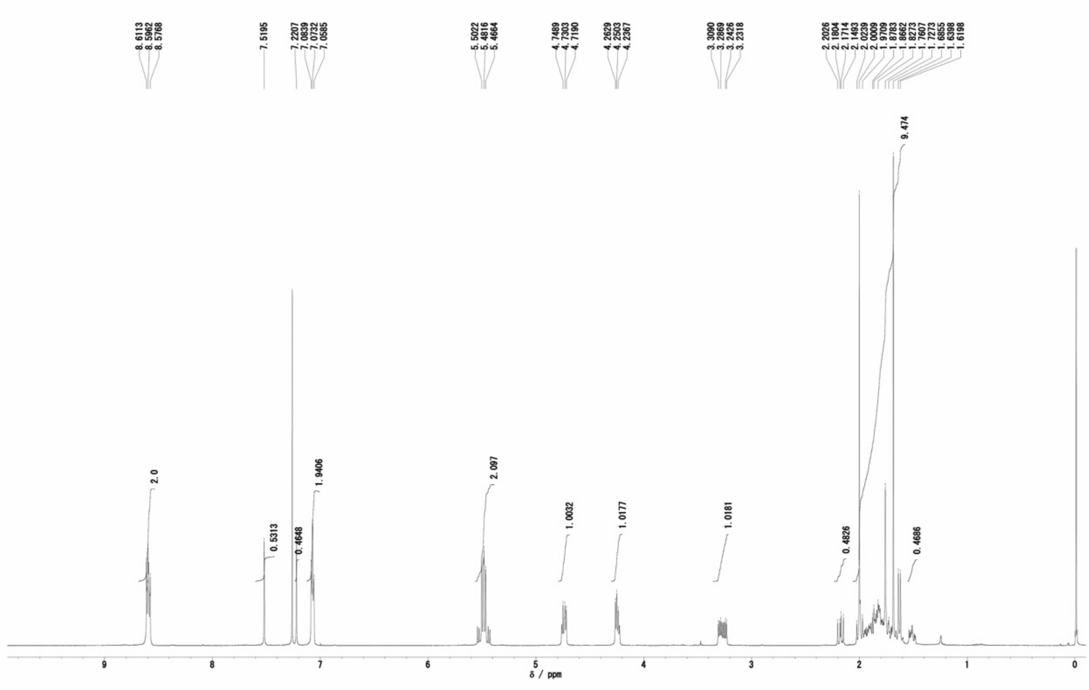

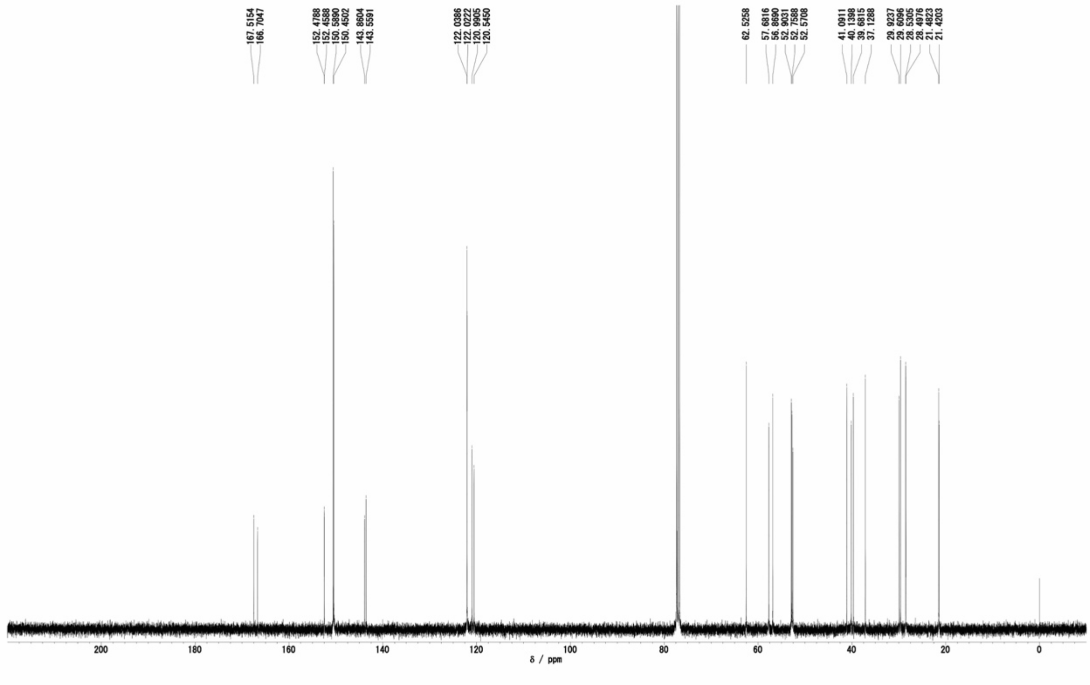

^1H (400 MHz) and ^{13}C (100 MHz) NMR spectra of ***endo*-7c** ($CDCl_3$)

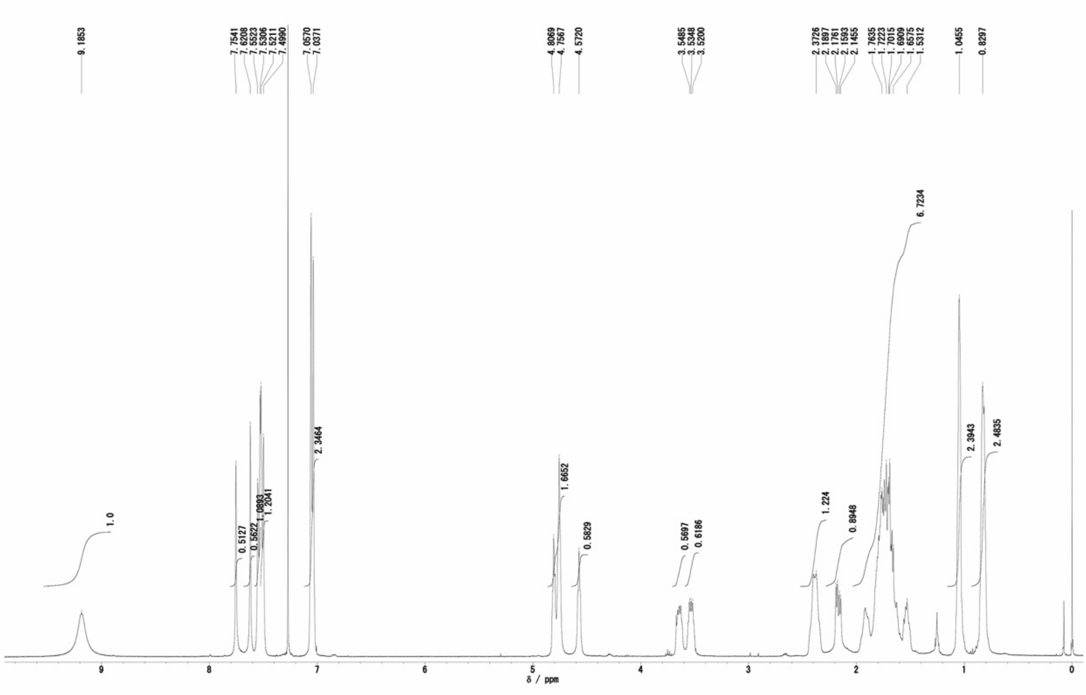

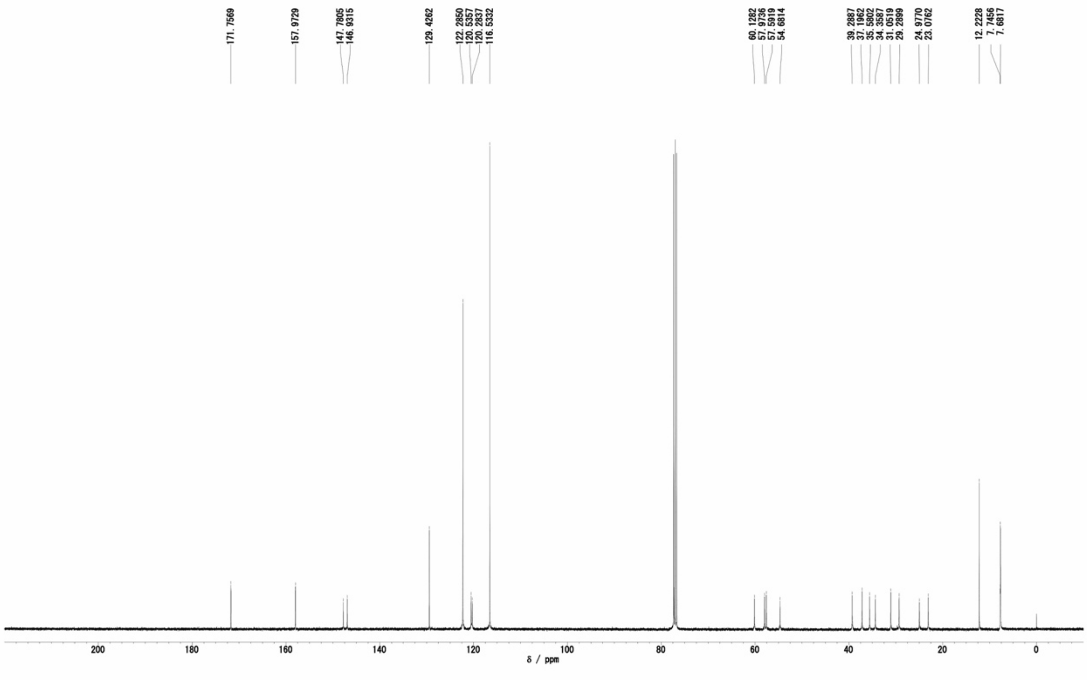

^{1}H (400 MHz) and ^{13}C (100 MHz) NMR spectra of ***exo*-7e** ($CDCl_3$)

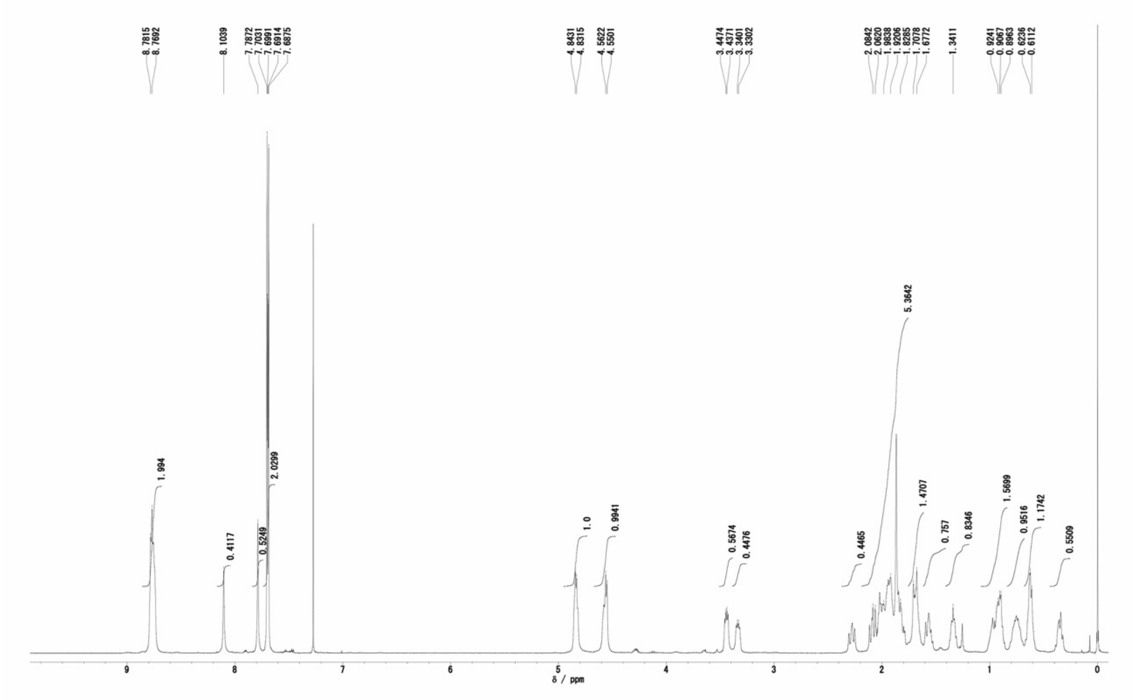

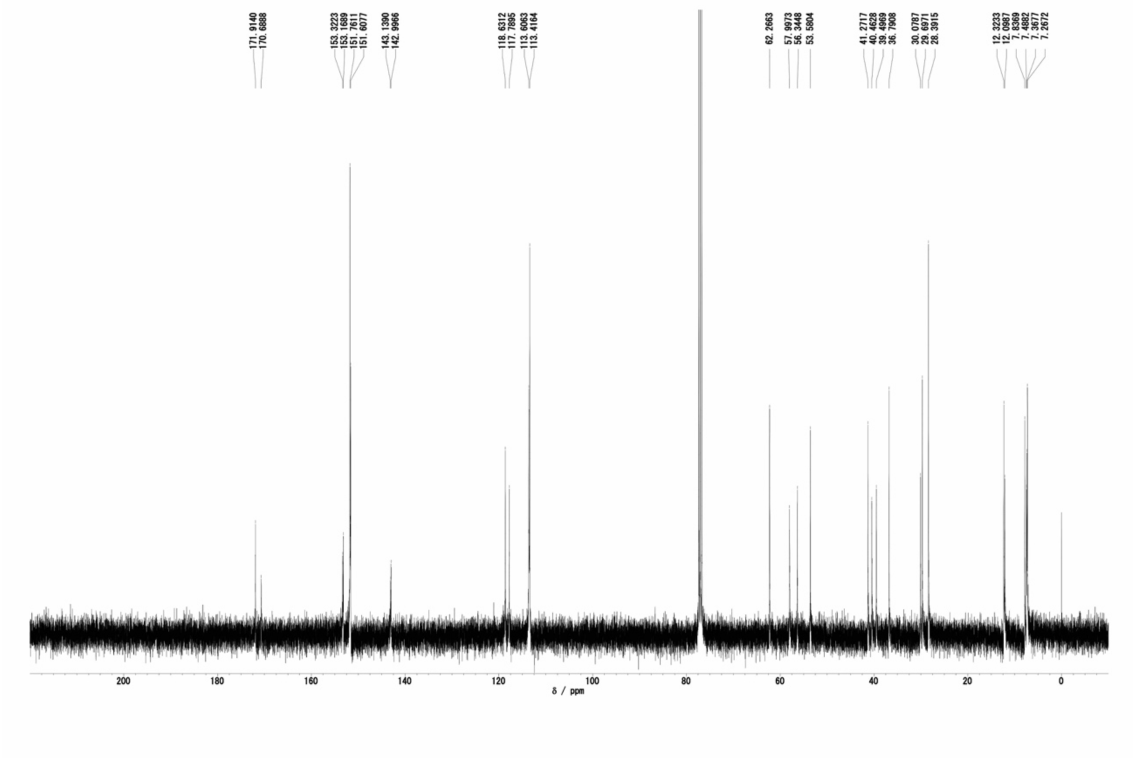

Supplement: Supplementary file 1 [file molecules-28-06925-s001.zip › molecules-2625888-supplementary.pdf]
